# Amlexanox reduces new-onset atrial fibrillation risk in sepsis by downregulating S100A12: a Mendelian randomization study

**DOI:** 10.3389/fcvm.2024.1401314

**Published:** 2024-10-09

**Authors:** Hang Yang, Lin Feng, Zhenjie Jiang, Xiaodan Wu, Kai Zeng

**Affiliations:** ^1^ Department of Anesthesiology, The First Affiliated Hospital of Fujian Medical University, Fuzhou, China; ^2^ Department of Hematology, The First Affiliated Hospital of Fujian Medical University, Fuzhou, China; ^3^ Department of Anesthesiology, Shengli Clinical Medical College, Fujian Medical University, Fuzhou, Fujian, China

**Keywords:** sepsis, atrial fibrillation, S100A12, amlexanox, inflammation, Mendelian randomization

## Abstract

**Background:**

Sepsis is characterized by high morbidity and mortality rates, alongside limited therapeutic efficacy. Atrial fibrillation (AF), the most common arrhythmia, has been closely linked to sepsis in prior research. However, the specific mechanisms through which sepsis leads to new-onset AF remain poorly understood. This study focuses on identifying critical genes that are dysregulated in the development of new-onset AF within the context of sepsis, with the goal of uncovering new potential targets for its diagnosis and prevention.

**Material and methods:**

Our study began by applying Mendelian Randomization (MR) to assess the causal link between sepsis and AF. We then sourced sepsis and AF datasets from the Gene expression Omnibus (GEO) database. Using Weighted Gene Co-expression Network Analysis (WGCNA), we pinpointed key modules and genes associated with both sepsis and AF conditions. Protein-protein interaction (PPI) network was constructed. The Transcriptional Regulatory Relationships Unravelled by Sentence-based Text-mining (TRRUST) database helped build the transcription factor (TF) interaction network. Key genes were scrutinized through Gene Ontology (GO), Kyoto Encyclopedia of Genes and Genomes (KEGG), Gene Set Enrichment Analysis (GSEA) and Gene Set Variation Analysis (GSVA) to delve into their roles in new-onset AF's pathophysiology during sepsis. We employed the CIBERSORT algorithm to evaluate immune infiltration and the association between key genes and immune cells. The Connectivity Map (CMap) database facilitated the prediction of potential small molecule compounds targeting key genes. To culminate, an acute sepsis mouse model was developed to validate the implicated mechanisms of key genes involved in new-onset AF during sepsis, and to assess the prophylactic effectiveness of identified drug candidates.

**Results:**

MR revealed potential independent risk factors for new-onset AF in sepsis. S100A12 was identified as a core interaction gene with elevated levels in sepsis and AF, underscoring its diagnostic and predictive significance. S100A12, along with associated genes, was mainly linked to immune and inflammatory response signaling pathways, correlating with immune cell levels. Targeting S100A12 identifies five potential small molecule therapeutics: amlexanox, balsalazide, methandriol, olopatadine, and tiboloe. In animal studies, acute sepsis increased S100A12 expression in serum and atrial tissues, correlating positively with inflammatory markers (IL-1β, IL-6, TNF-α) and negatively with heart rate, indicating a predisposition to AF. Early amlexanox administration can reduced S100A12 expression, dampened inflammation, and lessened new-onset AF risk in sepsis.

**Conclusion:**

This study demonstrates that sepsis may independently increase the risk of new-onset AF. We identified S100A12 as a key gene influencing the new-onset AF in sepsis through immune regulation, presenting considerable diagnostic and predictive value. Notably, amlexanox, by targeting S100A12 emerges as the most clinical relevant intervention for managing new-onset AF in sepsis patients.

## 1 Introduction

Sepsis, a systemic inflammatory response arising from severe infection, trauma, burns, shock, and surgery, is a significant health challenge worldwide. In China, it is estimated that there are over 9 million cases of severe sepsis annually, with an annual growth rate between 1.5% and 8.0%. This condition poses a grave threat, resulting in more than 800,000 deaths each year (1). Extensive research has established a strong connection between sepsis and various cardiovascular diseases, including arrhythmia, hypertension, coronary artery disease, heart failure, and stroke (2–6). Among these, atrial fibrillation (AF), a prevalent cardiac arrhythmia, is noteworthy for its rising incidence (7). AF significantly increases the risk of stroke and heart failure, thereby escalating both healthcare costs and societal economic burdens (8, 9). The link between sepsis and AF has garnered considerable attention, with studies showing a direct correlation between the severity of sepsis and the incidence of new-onset AF (10, 11), indicating a substantial risk of AF recurrence post-treatment (11). Shared risk factors between sepsis and AF, such as age, infection, obesity, metabolic disorders, and electrolyte imbalances, suggest a complex interplay (12–15). Despite known associations with inflammation, autonomic dysfunction, and oxidative stress, the precise mechanisms driving new-onset AF in sepsis remain elusive (16–18).

In this study, our goal was to clarify the causal link between sepsis and the development of AF using bioinformatics analysis, positing that dysregulation of key genes could underlie new-onset AF in sepsis. Furthermore, we aimed to experimentally investigate the potential mechanism by which S100A12 contributes to new-onset AF in sepsis, examining the relationship between S100A12 expression, inflammation and AF, and assessing the efficacy of potential therapeutic agents for treating sepsis-induced new-onset AF. This research could yield novel biomarkers for detecting new-onset AF in sepsis patients, offering insights into its molecular underpinnings and identifying promising treatment options.

## 2 Materials and methods

### 2.1 Data acquisition

**Figure 1:**
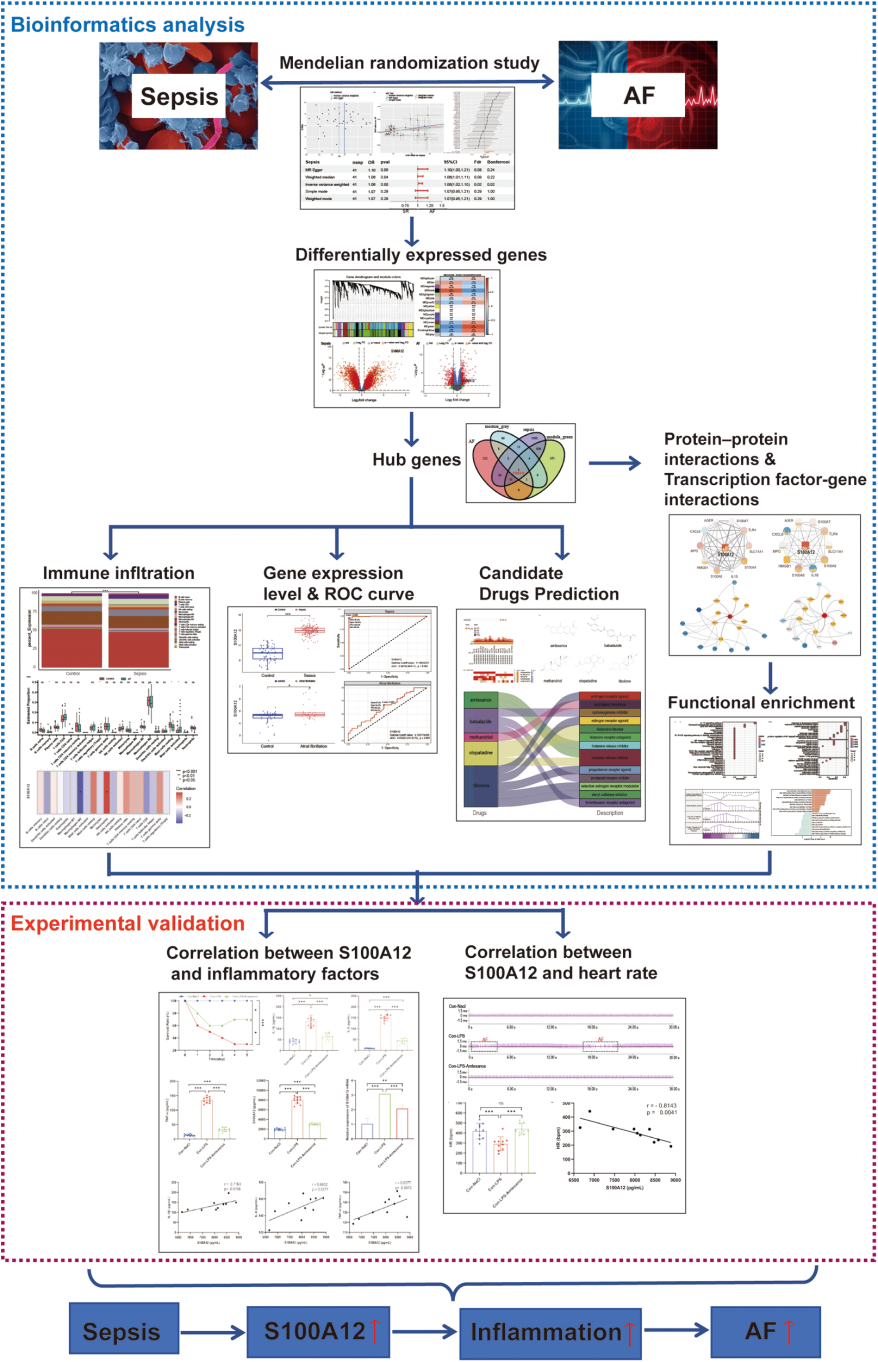
The flow chart of this study. AF, atrial fibrillation; ROC, operating characteristic curve.

In MR analysis, exposure data were sourced from the UK biobank database (https://www.ukbiobank.ac.uk). Outcome data were based on the study by Jonas B Nielsen et al. (19). We utilized the GPL570 platform for transcriptome data and conducted Weighted Gene Co-expression Network Analysis (WGCNA). Sepsis and AF datasets were downloaded from the Gene Expression Omnibus (GEO) database (https://www.ncbi.nlm.nih.gov/geo/) download sepsis datasets (GSE95233, GSE28750 and GSE57065) and AF datasets (GSE31821 and GSE79768). The comprehensive research methodology of this study is illustrated in Figure 1.

### 2.2 Data processing

To validate the causality between sepsis and AF with precision and credibility, we initially selected single nucleotide polymorphisms (SNPs) with *p*-values below 5 × 10^−8^ as instrumental variables (IVs), yet significant SNPs eluded us. We then opted for SNPs with *p*-values below 5 × 10^−5^ as IVs, acknowledging a potential decrease in average IVs validity but a significant boost in overall validity. To mitigate the dilution of MR results from a lowered SNP threshold, we imposed stricter criteria: linkage disequilibrium (LD) *r*^2^ < 0.0001, genetic distance >100,000 kb, *F*-value > 10 and Minor Allele Frequency (MAF) < 0.01. To assure result reliability, we corrected *p*-values using the false discovery rate (FDR) and Bonferroni correction. PhenoScanner (https://www.phenoscanner.medschl.cam.ac.uk) was employed to eliminate confounders. We also conducted the MR-Egger intercept test to assess SNPs horizontal pleiotropy and utilized the MR-PRESSO outlier test to exclude outlier variants, addressing heterogeneity and horizontal pleiotropy concerns. Finally, the refined SNPs set was then used in the MR analysis (see Supplementary Materials 1 and 2 for details).

For the RNA sequencing (RNA-seq) data analysis, we utilized the “sva” R package for normalization and preprocessing of the datasets. Whenever multiple probes mapped to the same gene, we calculated the average of these values to represent the gene's expression level. To ensure consistency across datasets, we eliminated batch effects, aligning the data distributions by standardizing medians, and harmonizing the means and variances (see Supplementary Materials 3 and 4 for details).

### 2.3 Mendelian randomization (MR) analysis

We employed five widely used methods for MR analysis: inverse-variance weighted (IVW), weighted mode, MR-Egger regression, weighted median estimator (WME), and MR Pleiotropy RESidual Sum and Outlier (MR-PRESSO). Our primary reliance was on the IVW method, complemented by the other four techniques for comprehensive analysis. Moreover, to ensure the robustness of our findings, we conducted multiple sensitivity analyses. Leave-one-out analyses were utilized to access if the causal inference was disproportionately influenced by a single SNP. Additionally, we applied the “TwoSampleMR” package to evaluate heterogeneity between IVs, considering *p* < 0.05 indicative of significant heterogeneity.

### 2.4 Weighted Gene Co-expression Network Analysis (WGCNA)

We applied WGCNA to identify significant modular genes associated with sepsis and AF. Utilizing the “WGCNA” R package, we constructed co-expression networks, setting the soft thresholding power *β* to an R2 cutoff value of 0.86 to ensure a scale-free network. The minimum module size was established at 30 genes. We calculated the correlation between various modules and the diseases, and produced a module-disease correlation heatmap. Criteria for gene significance (GS, |GS| > 0.5) and module membership (MM, |MM| > 0.8) were set to explore the association between modules and diseases. Modules showing the strongest correlations were identified, and their genes were selected as candidates for further analysis.

### 2.5 Identification of differentially expressed genes (DEGs)

For the identification of DEGs in sepsis and AF datasets, we employed the “Limma” R package for analysis. A screening criterion of |log2FC| > 0.232 and *p* < 0.05 was established. Volcano plot of DEGs were created using the “EnhancedVolcano” R package. Additionally, the “VennDiagram” R package was utilized to overlap the upregulated genes in the DEGs of sepsis and AF with the upregulated key module genes, aiming to pinpoint the core genes mediating the interaction between sepsis and AF.

### 2.6 Enrichment analysis

We analyzed and visualized Gene Ontology (GO) and Kyoto Encyclopedia of Genes and Genomes (KEGG) using the “clusterProfiler” and “ggplot2” R packages to access genes’ involvement in biological processes (BP), molecular functions (MF), cellular components (CC) and signaling pathways. Gene Set Enrichment Analysis (GSEA) was conducted with the “enrichplot” R package, adopting |NES| > 1, *p*.adjust < 0.05 and FDR < 0.25 as the screening criteria, highlighting the top five pathways. Furthermore, we utilized The Molecular Signatures Database (MSigDB) (https://www.gsea-msigdb.org/gsea/msigdb/index.jsp) to gather key gene-related sets, which were then analyzed with the “GSVA” R package. We focused on the top 10 signaling pathways, selecting those with logFC > 0 and logFC < 0 for detailed examination.

### 2.7 Immune infiltration analysis

We analyzed the abundance of immune cells in sepsis and AF using the “CIBERSORT” R package. Visualization of immune cell abundance and proportions in each sample was accomplished with the “ggplot2” package, using bar plots. Box plots depicted the abundance of each immune cell type in the disease vs. the control group, and differences were assessed using the Wilcoxon test, with *p* < 0.05 denoting statistical significance. Additionally, the “pheatmap” R package was employed to generate a heatmap, illustrating the association between key genes common to both sepsis and AF and the abundance of immune cells.

### 2.8 Receiver operating characteristic (ROC) curve

The “multipleROC” R package was utilized to generate the receiver operating characteristic (ROC) curve, calculate the area under the curve (AUC) along with its 95% confidence interval (CI), and assess the clinical diagnostic and predictive value.

### 2.9 Protein-protein interaction (PPI) network and transcription factor (TF) interaction network

We constructed the PPI network for targeted genes through the STRING database (https://string-db.org/), setting the confidence threshold at 0.4 for required interactions while keeping other parameters at their default settings. The TF interaction network was constructed using the Transcriptional Regulatory Relationships Unraveled by Sentence-based Text mining (TRRUST) (http://www.grnpedia.org/trrust). Subsequently, both the PPI and TF interaction networks were visualized using Cytoscape software (version 3.10.1).

### 2.10 Prediction of drug candidates

The Connectivity Map (CMap) database (https://www.c-map.com/) serves as a resource for linking gene expression profiles with disease states. By inputting our target genes into the CMap database, we matched them against the differential gene maps available. Based on the enrichment score ranking, we identified and screened the top five small molecule compounds that exhibited the highest similarity to our targeted gene loci.

### 2.11 Construction of mouse sepsis model

Healthy adult male mice (weight 25 ± 3 g) were randomly divided into three groups: a control saline group (Con-NaCl, *n* = 10), a control sepsis group (Con-LPS, *n* = 10), and an Amlexanox intervention group (Con-LPS-Amlexanox, *n* = 10), following the ethical guidelines for the care and use of laboratory animals. All animal procedures were approved by the Institutional Animal Care and Use Committee (IACUC) and conducted in accordance with the principles of animal welfare (No. FJMU IACUC 2021-0336). Lipopolysaccharide (LPS) (Sigma, L2880, USA) was prepared in normal saline at a concentration of 3 mg/ml, with each mouse in sepsis group receiving an intraperitoneal injection of 0.1 ml. The saline group received an equivalent volume of normal saline as an intraperitoneal injection. Prior to LPS injection, the Amlexanox intervention group was administered Amlexanox via oral gavage at a dosage of 100 mg per kilogram (MedChemExpress, 68302-57-8, USA), ensuring minimal stress and discomfort to the animals. The eligibility criteria for each group are shown in the Table 1.

**Table 1:** The eligibility criteria for each group.

Group	Eligibility criteria
Control saline group (Con-NaCl) (*n* = 10)	Healthy adult male mice; Weight: 25 ± 3 g; Age: 8–12 weeks; No prior history of infection, illness, or surgery; No prior exposure to LPS or Amlexanox; Normal activity levels and behavior observed.
Control sepsis group (Con-LPS) (*n* = 10)	Healthy adult male mice; Weight: 25 ± 3 g; Age: 8–12 weeks; No prior history of infection, illness, or surgery; Normal activity levels and behavior observed; No prior treatment with Amlexanox; Administered LPS via intraperitoneal injection at a dose of 0.3 mg/ml; No current medication or treatments affecting immune response.
Amlexanox intervention group (Con-LPS-Amlexanox) (*n* = 10)	Healthy adult male mice; Weight: 25 ± 3 g; Age: 8–12 weeks; No prior history of infection, illness, or surgery; Normal activity levels and behavior observed; No prior exposure to LPS; Received oral gavage of Amlexanox prior to LPS injection at a dosage of 100 mg/kg; Administered LPS via intraperitoneal injection at a dose of 0.3 mg/ml; No signs of distress or abnormal behavior post-treatment.

Con, control; LPS, Lipopolysaccharide.

### 2.12 Electrocardiograph (ECG) monitoring

Anesthesia was initiated in an induction box using a 3% sevoflurane and 30% oxygen mixture, delivered at a 0.5 ml/min flow rate. Subsequently, the mice were positioned in the supine on a constant temperature heating pad set to 37℃, with the sevoflurane concentration adjusted to 1%–2% to maintain anesthesia. The anesthetized mice were then connected to the MD3000 biosignal acquisition and processing system (Zhenghua Biological Instrument, Huaibei, China), allowing for the recording of a 5-min baseline ECG that was stable, clean, and free from interference.

### 2.13 Enzyme-linked ImmunoSorbent Assay (ELISA)

ELISA kits were employed to measure the serum levels of IL- 1β (Boster, EK0394, China, 12.5–800 pg/ml), IL-6 (Boster, EK0411, China, 15.6–1,000 pg/ml), TNF-α (Boster, EK0527, China, 15.6–1,000 pg/ml) and S100A12 (Feiya Biotechnology, 45996M2, China, 0.1–4.5μg/L) in mice. Experimental protocols were meticulously followed as per the manufacturer's guidelines, and concentration results were normalized using the provided kit reagents.

### 2.14 Reverse transcription quantitative polymerase chain reaction (RT-qPCR)

RT-qPCR was utilized to assess the mRNA expression levels of S100A12 in the atrial tissue across different groups. Total RNA was isolated using Trizol reagent, with samples subsequently separated into aqueous, intermediate, and organic layers upon chloroform addition. The RNA, collected from the aqueous layer and precipitated with isopropyl alcohol, was then reverse transcribed into cDNA using primers. This cDNA served as the template for PCR amplification. To confirm RNA integrity, the products underwent 1% agarose gel electrophoresis, and RT-qPCR identified the expression levels of the target gene. The reaction setup followed kit instructions precisely, with conditions including a pre-denaturation at 94°C for 1 min, denaturation at 94°C for 20 s, annealing at 60°C for 40 s, extension at 72°C for 40 s, over 40 cycles. Target gene mRNA expression levels were quantified using the 2^−△△CT^ method, with GAPDH as the internal control. This procedure was repeated thrice to calculate an average result. The S100A12 primer sequences were GGTGGTCATATGACAAAACTTGAAGAG and GGTGGTACTAGTGCATCTCCCGTGATGCACTCTTTGTGGGTGTGG, and the GAPDH primer sequences were ATGTTGCAACCGGGAAGGAA and ACGGACACCTAATCCTCCCA.

### 2.15 Statistic analysis

All bioinformatics analyses and visualizations were conducted using R software (version 4.31), with a *p*-value < 0.05 deemed statistically significant. For experimental verification, SPSS 26.0 was utilized for statistical analysis. Quantitative data were presented as mean ± standard deviation ($\bar{x}\pm s$). The *t*-test was applied for comparisons between two groups, while one-way analysis of variance (ANOVA) was used for comparing the means across multiple groups, followed by post-hoc comparisons using the least significant difference (LSD) *t*-test. *p*-value < 0.05 was considered statistically significant.

## 3 Results

### 3.1 Sepsis may serve as an independent risk factor for AF

**Figure 2:**
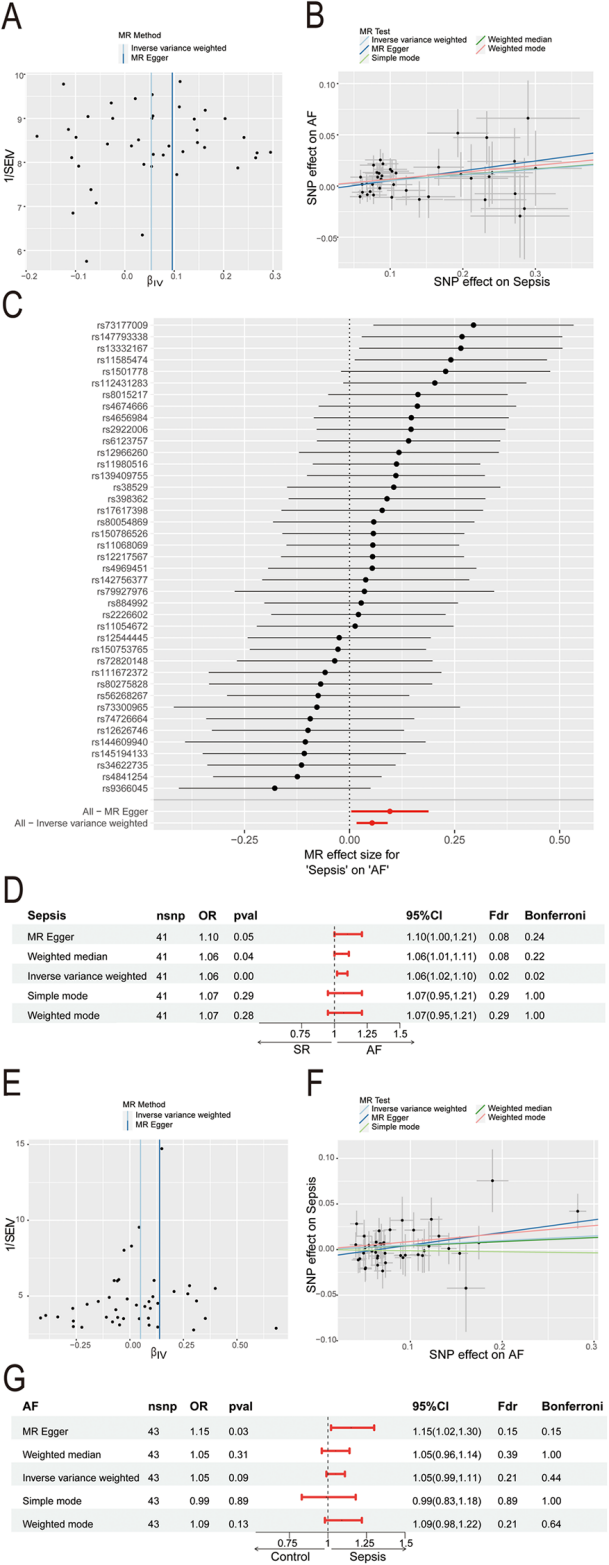
Mendelian Randomization study of sepsis and AF. **(A)** Funnel plot of SNP heterogeneity in sepsis; **(B)** scatter plot of causal estimates of sepsis on AF under different MR Methods; **(C)** leave-one-out sensitivity analysis of the association between sepsis and atrial fibrillation; **(D)** estimating the causal effect of sepsis on AF under different MR Methods; **(E)** funnel plot of SNP heterogeneity in AF; **(F)** scatter plot of causal estimates of AF on sepsis under different MR Methods; **(G)** estimating the causal effect of AF on sepsis under different MR Methods; AF, atrial fibrillation; MR, Mendelian randomization; SNP, single nucleotide polymorphisms; CI, confidence interval; OR, odds ratio.

The sepsis data included 486,484 participants with 11,643 sepsis cases and 12,243,539 associated SNPs. A total of 1030,836 participants and 60,620 AF cases were included in the AF data. In the sepsis analysis, confounder rs13152538 was removed, due to its association with coronary artery disease, which could skew causality analysis between sepsis and AF. We ultimately selected 41 SNPs with minimal heterogeneity among them (Figure 2A). Sepsis was positively associated with the risk of AF (Figure 2B). The “leave-one-out” sensitivity analysis confirmed the robustness of the MR results (Figure 2C). Cochran's *Q* test revealed no evidence of heterogeneity (Cochran's *Q* = 41.52, *P* = 0.40), and the MR-Egger intercept indicated no evidence of horizontal pleiotropy (*P* = 0.33). The results of MR indicated a potential causal relationship between sepsis and atrial fibrillation (OR = 1.06, 95% CI: 1.02–1.10, *P* = 0.004) (Figure 2D). However, the results of reverse MR indicated no significant causal relationship between AF and sepsis (OR = 1.05, 95% CI: 0.99–1.11, *P* = 0.09) (Figures 2E–G).

### 3.2 S100A12 represents a key shared gene between sepsis and AF

**Figure 3:**
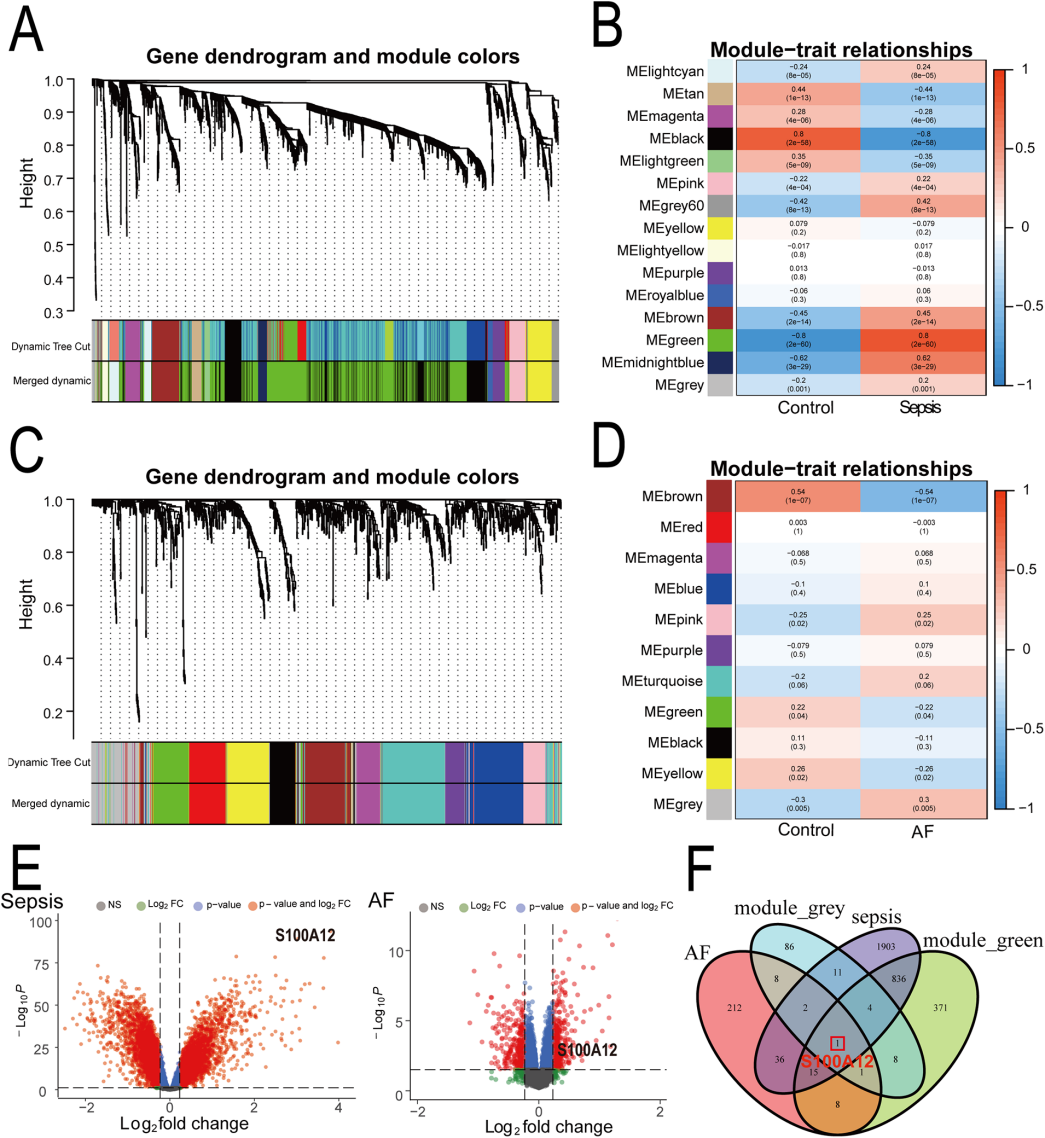
Identification of shared core genes for sepsis and AF using WGCNA. **(A)** Cluster dendrogram of co-expressed genes in sepsis; **(B)** heatmap of module-trait relationships in sepsis; **(C)** cluster dendrogram of co-expressed genes in AF; **(D)** heatmap of module-trait relationships in AF; **(E)** volcano plot for DEGs in the sepsis and AF datasetst; **(F)** Venn plot of the upregulated DEGs in sepsis and AF intersecting significant module genes in sepsis and AF; AF, atrial fibrillation; DEGs, differentially expressed genes; WGCNA, weighted gene co-expression network analysis.

Through WGCNA analysis of the sepsis dataset, we identified 15 gene modules significantly linked with sepsis progression, compared to normal blood samples. These modules are each denoted by a distinct color. Notably, the “MEgreen” module (comprising 1,244 genes, *r* = 0.80, *P* = 2e−60) exhibited the strongest positive correlation with sepsis (Figures 3A–B, Supplementary Material 5). Similarly, analysis of the AF dataset revealed 11 modules, with the “MEgrey” module (comprising 121 genes, *r* = 0.30, *P* = 0.005) demonstrating the most significant positive association with AF (Figures 3C–D, Supplementary Material 6). Consequently, we selected the “MEgreen” module from sepsis analysis and the “MEgrey” module for the AF analysis as our focus. Subsequent screening identified 6,189 DEGs in the sepsis dataset, including 2,808 up-regulated genes and 3,381 down-regulated genes (Figure 3E, Supplementary Material 7). In the AF dataset, We found 514 DEGs, with 283 up-regulated and 231 down-regulated genes (Figure 3E, Supplementary Material 8). Ultimately, intersecting the “MEgreen” module related to sepsis and the “MEgrey” module associated with AF with their respective up-regulated genes highlighted S100A12 as the core shared gene between sepsis and AF (Figure 3F).

### 3.3 Biological processes and signaling pathways related to S100A12

**Figure 4:**
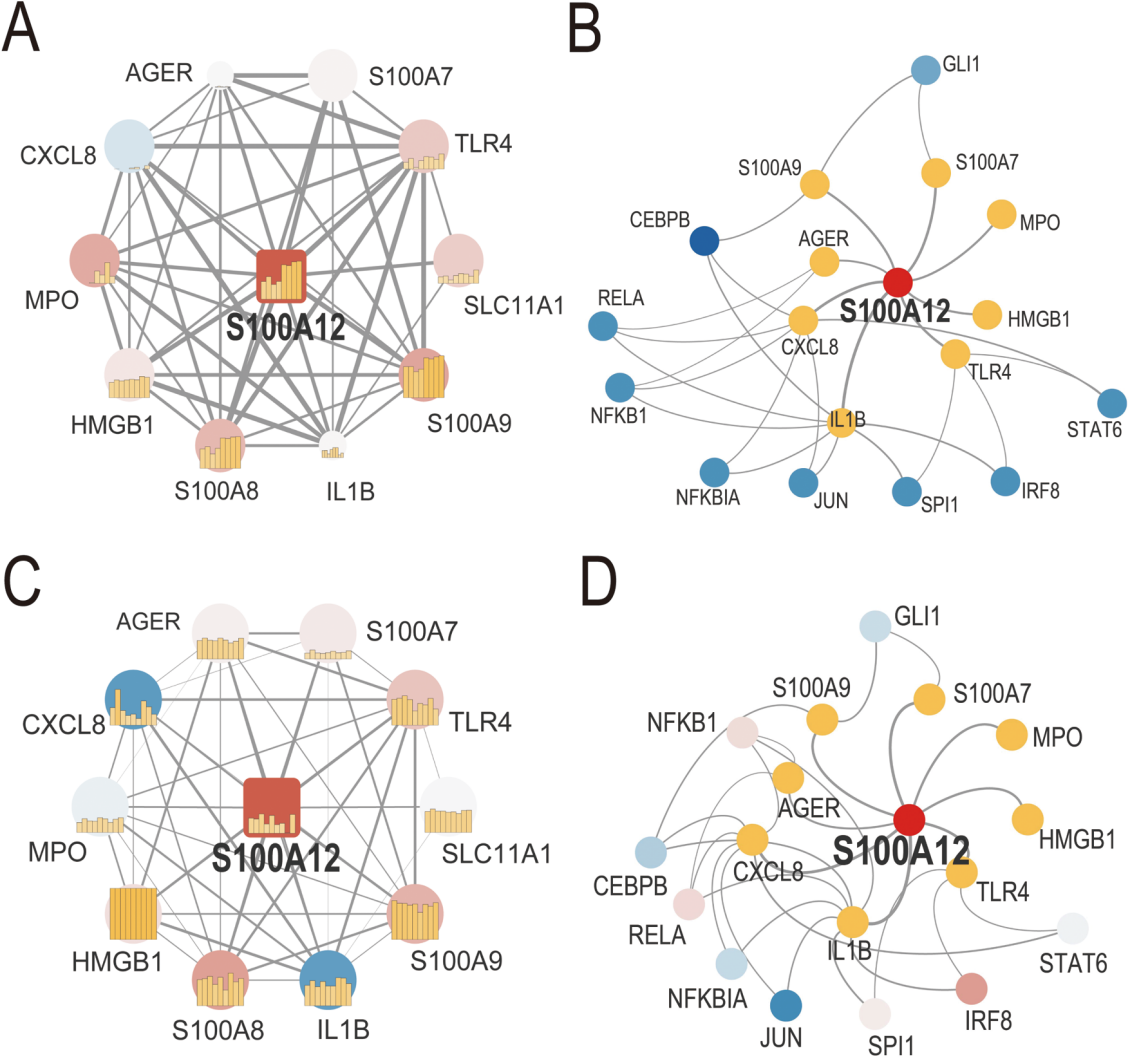
PPI network and TF-gene interaction network of S100A12 and related genes in sepsis and AF. **(A)** PPI network of S100A12 and its interacting proteins in sepsis; **(B)** TF-gene interaction network of S100A12 and its interacting genes in sepsis; **(C)** PPI network of S100A12 and its interacting proteins in AF; **(D)** TF-gene interaction network of S100A12 and its interacting genes in AF; PPI, protein-protein interaction; TF, transcription factor.

We developed S100A12-related PPI networks in sepsis and AF, identifying S100A12 and its associated proteins and analyzing their expression levels (Figures 4A,C, Supplementary Materials 9, 10). In sepsis, significant upregulation was observed for S100A12, TLR4, SLC11A1, S100A9, S100A8, S100A7, HMGB1, and MPO, while CXCL8 showed downregulation. AGER and IL1B expressions remained relatively unchanged (Figure 4A, Supplementary Material 9). In AF, S100A12, TLR4, S100A9, and S100A8 were up-regulated, and IL1B was down-regulated. Expressions of SLC11A1, HMGB1, AGER, S100A7, CXCL8, and MPO did not show significant changes (Figure 4C, Supplementary Material 10). Furthermore, TF-gene interaction networks for S100A12 and related genes in sepsis and AF were constructed (Figures 4B,D, Supplementary Materials 11, 12). In sepsis, RELA, CEBPB, NFKBIA, STAT6, IRF8 and SPI1 were downregulated, with no significant change in NFKB1, JUN and GLI1 (Figure 4B, Supplementary Material 11). In AF, IRF8 was upregulated, JUN was downregulated, and GLI1, STAT6, SPI1, NFKBIA, GLI1, STAT6, SPI1, NFKBIA. NFKB1, RELA and CEBPB showed no significant change (Figure 4D, Supplementary Material 12).

**Figure 5:**
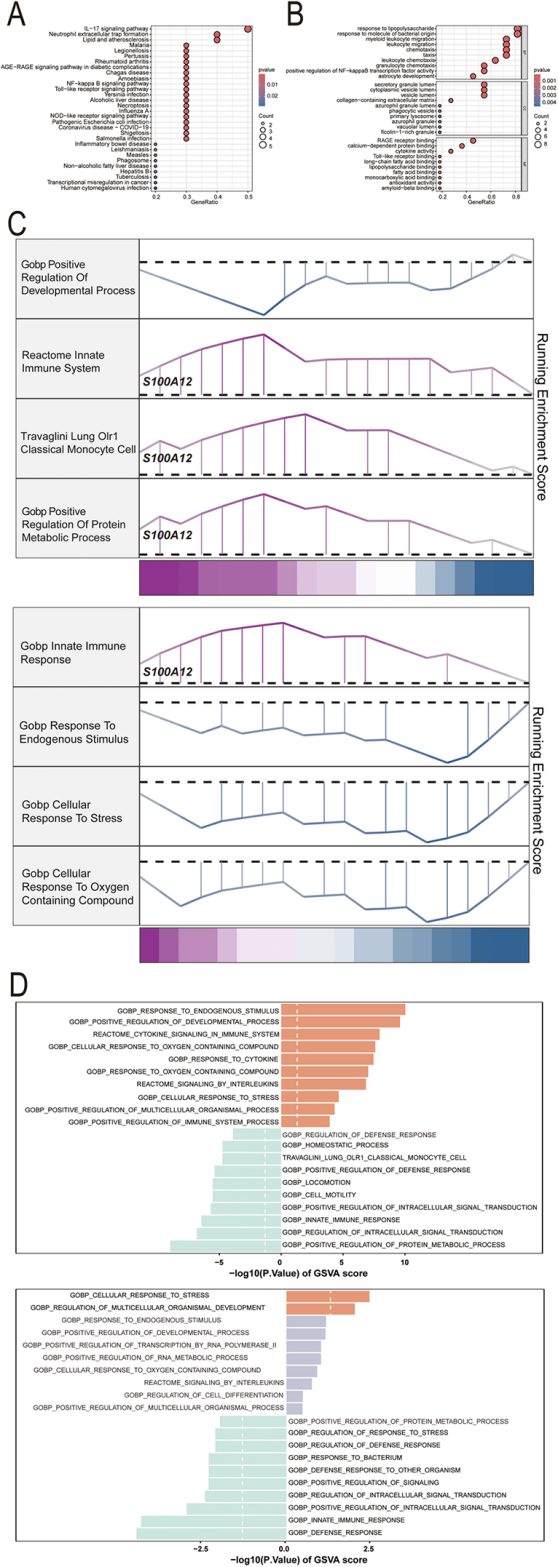
The selected nodes of S100A12 from PPI network were used for functional enrichment analysis. **(A)** Results of KEGG pathway analysis of S100A12 and its interacting proteins; **(B)** results of GO enrichment analysis of S100A12 and its interacting proteins; **(C)** pathways enriched for GSEA in sepsis and AF patients with high S100A12 expression; **(D)** GSEA of the top 4 enriched pathways in sepsis and AF patients with high S100A12 expression; **(E)** GSVA of the top 10 enriched pathways in sepsis and AF patients with high S100A12 expression; AF, atrial fibrillation; DEGs, differentially expressed genes; GO, Gene Ontology; KEGG, Kyoto Encyclopedia of Genes and Genomes; BP, biological process; CC, cellular component; MF, molecular function. KEGG, Kyoto Encyclopedia of Genes and Genomes; GSEA, Gene Set Enrichment Analysis; GSVA, gene set variation analysis.

GO, KEGG pathway, GSEA and GSVA methods were utilized to delve into the molecular mechanism of S100A12 and related genes (Figures 5A–D). GO analysis revealed enrichment in “response to lipopolysaccharide” and “response to molecule of bacterial origin” (BP), “secretory granule lumen”, “cytoplasmic vesicle lumen” and “vesicle lumen” (CC) and “RAGE receptor binding” (MF) (Figure 5B, Supplementary Material 13). KEGG pathway analysis linked S100A12 and related genes with the “IL-17 signaling pathway” (Figure 5A, Supplementary Material 14). GSEA highlighted enrichment in “innate immune system”, “lung olr1 classical monocyte cell” and “positive regulation of protein metabolic process” pathways in sepsis (Figure 5C, Supplementary Material 15), and the “innate immune response” pathway in AF (Figure 5C, Supplementary Material 16). GSVA in sepsis showed enrichment in “response to endogenous stimulus” and “positive regulation of developmental process” pathways, whereas in AF, the focus was on “cellular response to stress” and “regulation of multicellular organismal development” pathways (Figure 5D, Supplementary Materials 17, 18).

### 3.4 S100A12 expression correlates with the abundance of immune cell in both sepsis and AF

**Figure 6:**
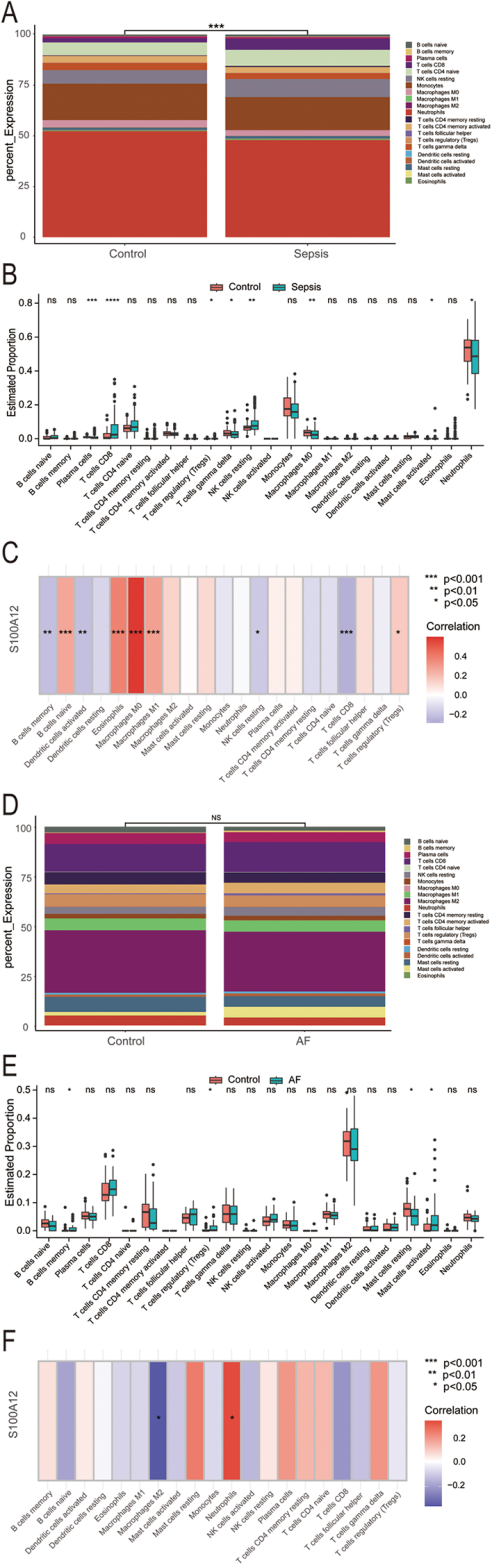
Analysis of immune infiltration in sepsis and AF. **(A)** Stacked histogram of the proportion of immune cells in sepsis and control group; **(B)** boxplot of the expression of 22 immune cells in sepsis and controls; **(C)** heat map of correlation between S100A12 expression levels and immune cells in sepsis; **(D)** stacked histogram of the proportion of immune cells in AF and control group; **(E)** boxplot of the expression of 22 immune cells in AF and controls; **(F)** heat map of correlation between S100A12 expression levels and immune cells in AF; ns, no significance, *P < 0.05, **P < 0.01, and ***P < 0.001.

Our analysis revealed that S100A12 and its associated genes predominantly influence inflammation and immune response. In the sepsis group, there was a notable variance in 22 immune cell compositions compared to the control group (*P* < 0.001) (Figure 6A). Relative to the control group, increases were observed in the abundance of CD8T cells (*P* < 0.0001), resting NK cells (*P* < 0.01), regulatory T cells (Tregs) (*P* < 0.05), and activated mast cells (*P* < 0.05) within the sepsis group. Conversely, decreases were seen in plasma cells (*P* < 0.001), gamma delta T cells (*P* < 0.05), M0 macrophages (*P* < 0.01), neutrophils (*P* < 0.05), with the remainder of immune cells showing no significant statistical difference (Figure 6B). S100A12 expression was positively correlated with the abundance of naive B cells (*r* = 0.266, *P* < 0.001), eosinophils (*r* = 0.379, *P* < 0.001), M0 macrophages (*r* = 0.586, *P* < 0.001), M1 macrophages (*r* = 0.287, *P* < 0.001) and Tregs (*r* = 0.159, *P* < 0.05) in sepsis. In sepsis, S100A12 expression level was negatively correlated with the abundance of memory B cells (*r* = −0.213, *P* < 0.01), activated dendritic cells (*r* = −0.214, *P* < 0.01), resting NK cells (*r* = −0.179, *P* < 0.05) and CD8T cells (*r* = −0.263, *P* < 0.001) (Figure 6C). No significant differences were found in the composition of 22 immune cells in the AF group compared to the control group (*P* = 0.83) (Figure 6D). In the AF group, the abundance of memory B cells (*P* < 0.05), Tregs (*P* < 0.05) and activated mast cells (*P* < 0.05) increased compared to the control, while the abundance of resting mast cells decreased (*P* < 0.05), with no statistical difference observed in the remaining immune cells (Figure 6E). In AF, S100A12 expression was positively correlated with neutrophils abundance (*r* = 0.347, *P* < 0.05) and negatively correlated with M2 macrophages abundance (*r* = −0.382, *P* < 0.05) (Figure 6F).

### 3.5 S100A12 expression is elevated in sepsis and AF, indicating diagnostic significance

**Figure 7:**
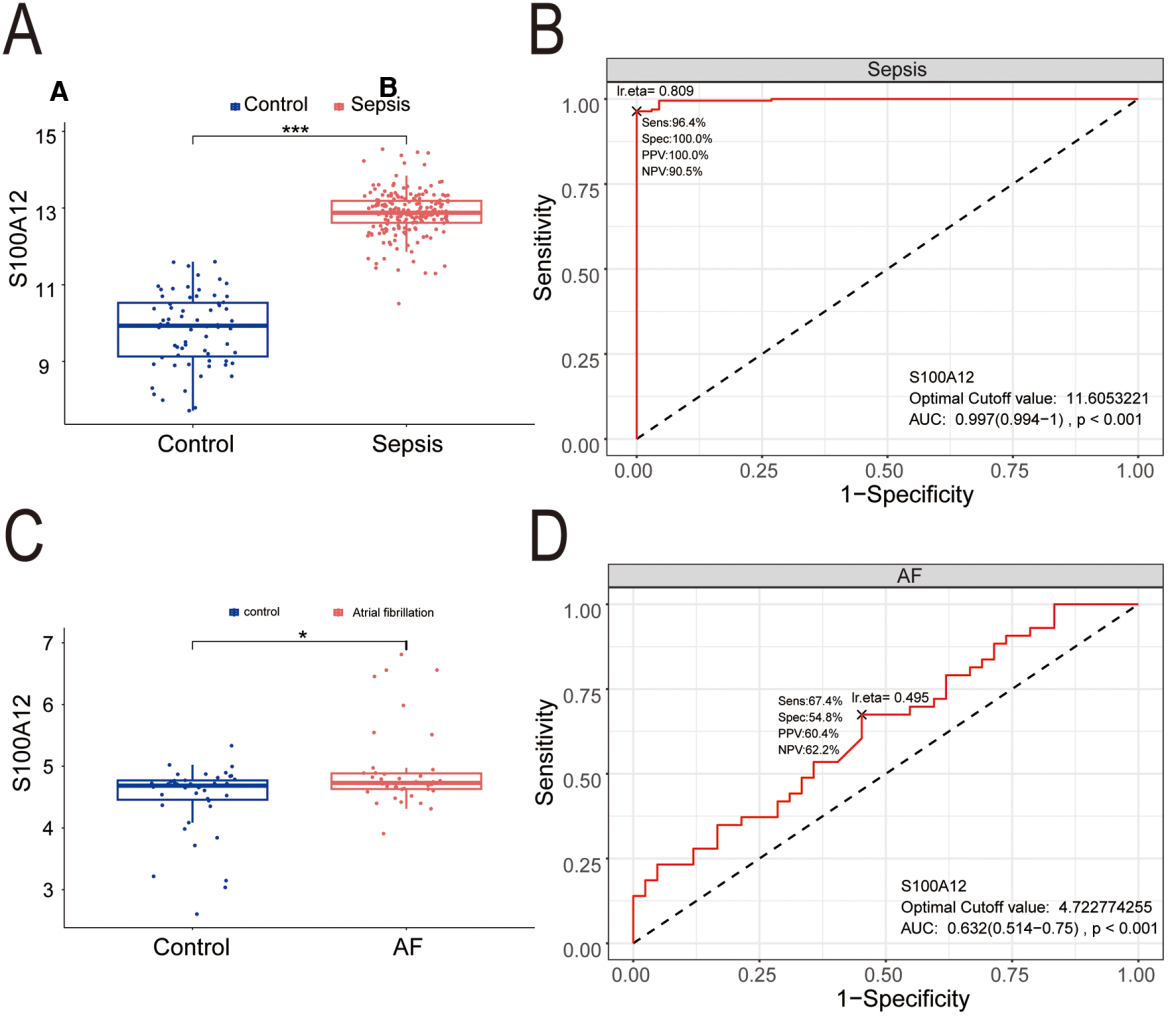
The expression level and diagnostic value of S100A12. **(A)** S100A12 expression level in sepsis and control group; **(B)** diagnostic value of S100A12 in sepsis; **(C)** S100A12 expression level in AF and control group; **(D)** diagnostic value of S100A12 in AF. AF, atrial fibrillation; AUC, area under the curve. *P < 0.05, **P < 0.01, and ***P < 0.001.

Compared to the control group, S100A12 expression was markedly higher in the sepsis group (*P* < 0.001) (Figure 7A), demonstrating significant diagnostic value for sepsis with an AUC of 0.997 (95% CI: 0.997–1.000, *P* < 0.001) (Figure 7B). S100A12 levels were elevated in the AF group compared to the controls (*P* < 0.05) (Figure 7C), indicating diagnostic relevance in AF with an AUC of 0.632 (95% CI: 0.514–0.750, *P* < 0.001) (Figure 7D).

### 3.6 Potential candidate small molecule compounds targeting S100A12 for treating new-onset AF in sepsis

**Figure 8:**
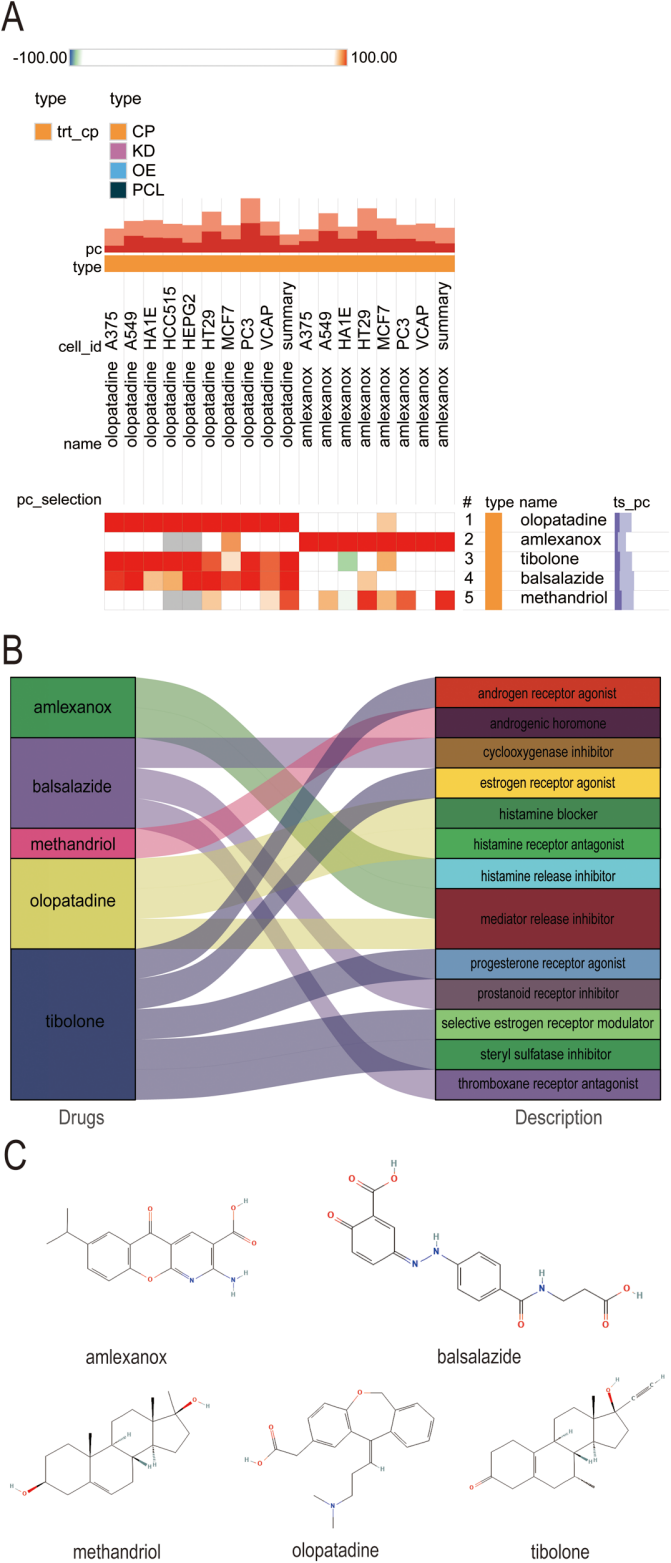
Potential small-molecule compounds targeting S100A12 for the treatment of new-onset AF in sepsis. **(A)** The heatmap of the top 5 compounds with the highest negative enrichment scores in 18 cell lines; **(B)** the description of the top 5 compounds; **(C)** the chemical structures of tthe top 5 compounds. cMAP, connectivity map.

Exploring the potential for small molecule drugs to treat new-onset AF in sepsis, we utilized the cMAP database to identify small molecules highly similar to S100A12 gene loci by enrichment score. These include amlexanox. balsalazide, methandriol, olopatadine and tibolone, pinpointed as prospective treatments targeting S100A12 for sepsis-related AF (Figure 8A). Thepathways targeted and chemical structures of these five compounds are detailed in Figures 8B,C, respectively.

### 3.7 S100A12 correlates with inflammatory markers and heart rate (HR), and amlexanox may lower the risk of new-onset AF in sepsis

**Figure 9:**
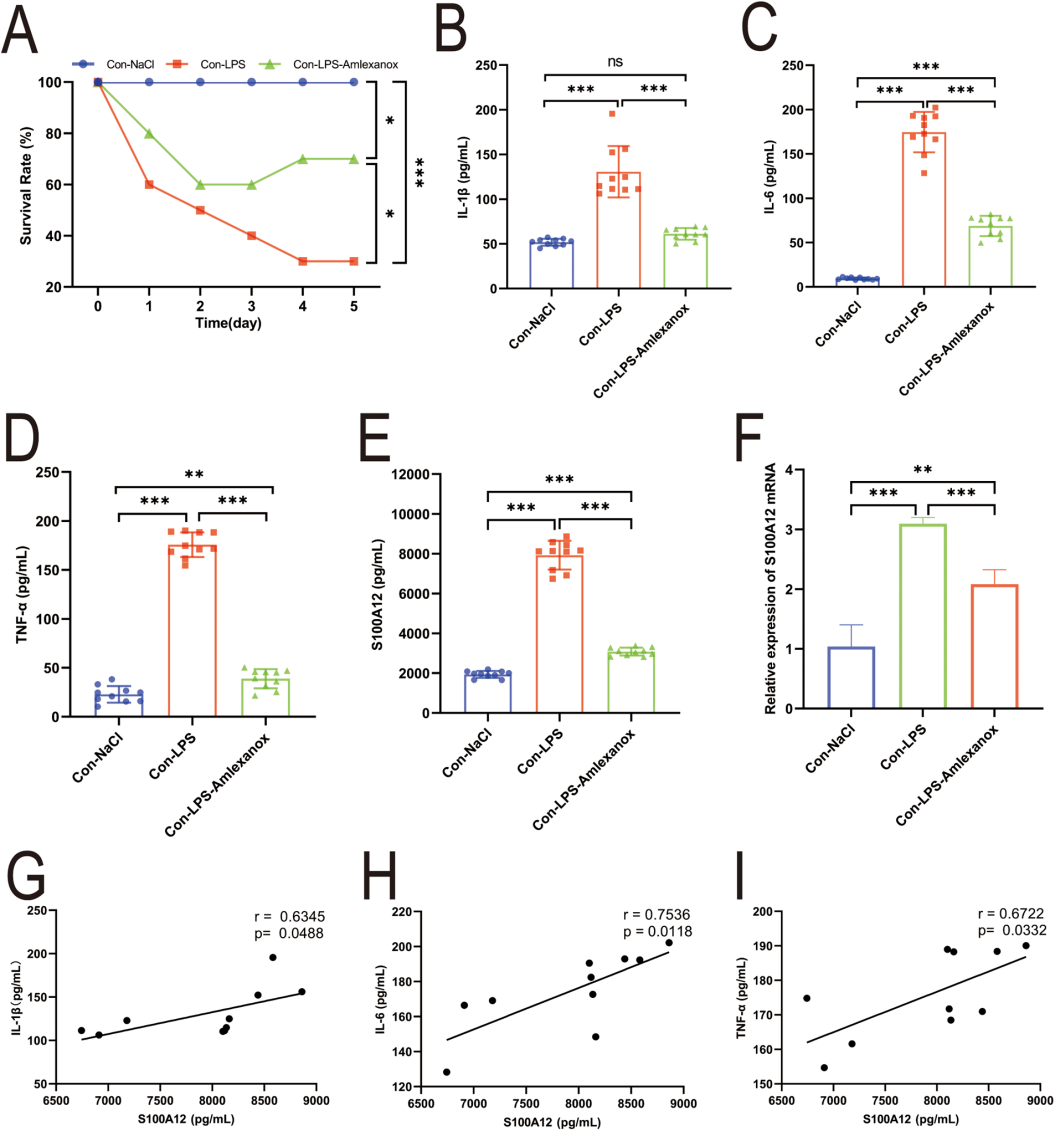
Correlation between S100A12 expression and inflammatory factors in mice with acute sepsis. **(A)** The five-day survival rate of mice with acute sepsis; **(B**–**D)** expression of inflammatory factors in serum of mice with acute sepsis **(E)** S100A12 expression in serum of mice with acute sepsis; **(F)** S100A12 gene expression in atrial tissue of mice with acute sepsis; **(G–I)** correlation between S100A12 expression and inflammatory factors in serum of mice with acute sepsis. Con, Control; LPS, Lipopolysaccharide, *P < 0.05, **P < 0.01, and ***P < 0.001.

**Figure 10:**
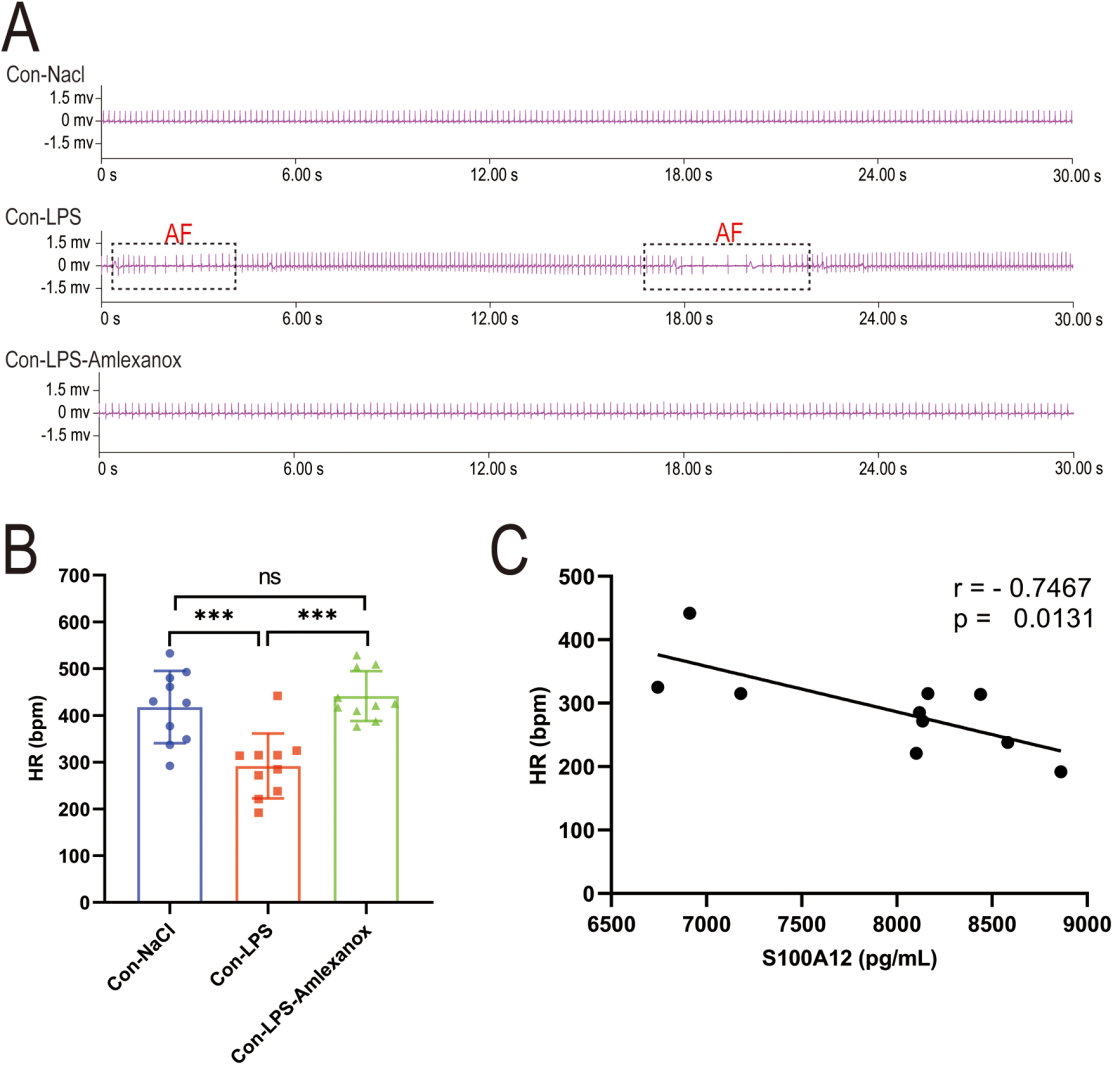
Correlation between S100A12 expression and heart rate in mice with acute sepsis. **(A)** Cardiac rhythm changes in acute septic mice; **(B)** heart rate in mice with acute sepsis; **(C)** correlation between S100A12 expression in serum and heart rate in mice with acute sepsis. Con, Control; LPS, Lipopolysaccharide; AF, atrial fibrillation; HR, heart rate, *P < 0.05, **P < 0.01, and ***P < 0.001.

The survival analysis of mice with acute sepsis indicated a high mortality rate associated with sepsis, while treatment with amlexanox contributed to a reduction in mortality (Figure 9A). Elisa results demonstrated a significant increase in both inflammatory markers and S100A12 expression in the serum of mice with acute sepsis; however, treatment with amlexanox was able to suppress the inflammatory response and reduce S100A12 expression (Figures 9B–E). Moreover, an upregulation in the mRNA expression of S100A12 was observed in the atrial tissues of these acute septic mice, which was mitigated by amlexanox treatment (Figure 9F). Correlation analysis further revealed a positive relationship between S100A12 expression and the levels of inflammatory markers (Figures 9G–I). ECG monitoring highlighted the presence of multiple spontaneous episodes of AF in mice with acute sepsis (Figure 10A), along with a significant decrease in HR (Figure 10B). Notably, there was an inverse correlation between S100A12 expression and HR (Figure 10C), and administration of amlexanox effectively reduced the risk of developing new-onset AF in sepsis (Figure 10).

## 4 Discussion

Numerous clinical studies have identified various risk factors for new-onset AF in sepsis, including age, infection, obesity, and electrolyte imbalances (10, 12, 14). Beyond these common pathogenic risks, genetic variations are increasingly recognized as critical in the pathogenesis of new-onset AF in sepsis. Large-scale genome-wide analysis have highlighted genetic variations’ significant role in both the emergence and progression of sepsis (20, 21). Similarly, a substantial evidence supports genetic factors’ vital contribution to the onset and persistence of AF (22–24). Genetic and bioinformatics studies offer methodologies to pinpoint independent genetic risk factors for diseases (25, 26). Yet, bioinformatics analysis specifically addressing new-onset AF in sepsis have been lacking. In our research, bioinformatics analysis helped us to identify sepsis as an independent risk factor for new-onset AF and to pinpoint S100A12 as a potential susceptibility gene. We also discovered potential small molecule drugs targeting S100A12 for treating new-onset AF in sepsis. By using an acute sepsis mouse model, we further established S100A12 as a key gene for new-onset AF in sepsis and examined amlexanox's impact on the S100A12 gene in treating this condition, underscoring S100A12's role in augmenting AF risk through inflammation promotion.

Numerous clinical studies have indicated that sepsis frequently leads to new-onset AF (11, 27, 28), yet the underlying mechanisms remain partially understood. Sepsis triggers systemic inflammation, activating various inflammatory factors (29–31). There's growing evidence that inflammation significantly contributes to the pathophysiology of AF (32–34), with inflammatory mediators altering atrial electrophysiology and structural substrates. This leads to atrial remodeling, thereby heightening AF susceptibility (32, 35–37). Additionally, inflammatory factors are known to affect calcium homeostasis and connexin levels in cardiomyocytes, causing atrial conduction abnormalities and electrical conduction instability (38, 39). Studies, such as those by Klein et al. and Zhao et al., have underscored AF as a common sepsis complication linked with increased mortality and have shown interventions like dapagliflozin can mitigate LPS-induced myocardial injury and AF susceptibility (40, 41). In addition, Aoki et al. (42) further revealed that in a sepsis model, the action potential duration (APD) in atrial myocytes is significantly shortened with cardiac ion channels dysfunctions contributing to AF development. Our MR analysis corroborates sepsis as an independent risk factor for new-onset AF, without reverse causality between the two. We also observed elevated inflammatory markers in the serum of mice with acute sepsis and noted spontaneous AF occurrences, suggesting a close link between new-onset AF in sepsis and an excessive inflammatory response. Thus, aligning with previous findings, our study proposes that the systemic inflammation characteristic of sepsis may drive new-onset AF.

This study predominantly utilized the highly reliable WGCNA algorithm to conduct co-expression cluster analysis on sepsis and AF datasets, ultimately identifying S100A2 as the core gene shared by sepsis and AF. The S100 calcium-binding protein A12 (S100A12) engages with cell surface receptors to trigger inflammatory signals, induce cytokine expression, and participate in the inflammatory response and immune regulation, playing a vital role in combating microbial infections and maintaining immune homeostasis (43–45). Additionally, S100A12 exhibits various extracellular activities, activating intracellular signaling cascades that produce cytokines, induce oxidative stress, and exacerbate the inflammatory response (46–49). Huang et al. observed a significant increase in S100A12 and inflammatory factor expression in LPS-induced septic rats, noting that S100A12 expression inhibition could reduce LPS-induced inflammatory factor levels (50). Our immune infiltration analysis revealed a correlation between S100A12 expression levels and immune cell abundance. Moreover, S100A12 and its associated genes were predominantly involved in inflammatory and immune pathways, underscoring its role in inflammatory and immune regulation, aligning with prior findings. Experimental validation showed increased S100A12 expression in the serum and atrial tissue of mice with acute sepsis, with a positive correlation between serum S100A12 expression and inflammatory markers, suggesting S100A12's potential as a biomarker for predicting inflammatory response.

Reports have highlighted the significance of serum S100A12 levels in diagnosing, predicting, and prognosticating cardiovascular diseases, positioning it as a novel biomarker for forecasting cardiovascular events (43, 51, 52). He et al. observed elevated levels of S100A12 and high-sensitivity C-reactive protein (HS-CRP) in chronic heart failure (CHF) patients’ plasma (53), underscoring S100A12 as a potential CHF biomarker with predictive value for cardiovascular events. Buyukterzi et al. proposed that the increased serum S100A12 in acute coronary syndrome (ACS) patients might indicate coronary plaque instability, playing a crucial role in ACS management (54). Zhang et al. established S100A12 as a superior marker for identifying ST-segment elevation myocardial infarction (STEMI), especially within the first 2 h post-symptom onset, and as a strong 1-year prognosis predictor in STEMI patients (55). The role of S100A12 in arrhythmias, however, remains less clear, with further exploration needed to understand its relationship with arrhythmia fully. This study confirmed S100A12 elevation in new-onset AF in sepsis. Our findings also linked S100A12 serum expression in acute sepsis mice with HR, suggesting that its increased expression may significantly reduce HR. This implies S100A12's involvement in new-onset AF's pathogenesis in sepsis, marking it as a critical regulatory gene. Hence, S100A12 offers significant clinical diagnostic value in sepsis and AF, emphasizing its importance in diagnosing, predicting, and prognosticating new-onset AF in sepsis.

As science and technology advance and focus on human health intensifies, drug research and development have emerged as a vibrant area of interest. Small molecule drugs, in particular, have gained prominence in therapeutic fields due to their distinct mechanisms of action and efficacy. Small molecule drugs are characterized by excellent tissue permeability, high oral bioavailability, uniform biodistribution, and metabolic stability. Their ease of synthesis further positions them favorably in drug development efforts. However, the treatment of new-onset AF in sepsis remains challenged by a lack of effective drugs, with no studies on high-throughput screening of potential small molecule therapeutics for this condition. Identifying potential small molecule compounds for treating new-onset AF in sepsis, based on crucial gene expression signatures shared between sepsis and AF, is critically needed. In this research, analysis of S100A12 via cMAP led to the identification of five small molecular compounds (amlexanox, balsalazide, methandriol, olopatadine, tibolone) as potential treatment candidates. Amlexanox stands out for its anti-inflammation, anti-allergy properties, ability to inhibit histamine and leukotriene production, and mast cells stabilization, primarily used in managing bronchial asthma and allergic rhinitis (56, 57). Increasingly, amlexanox is being recognized for its potential in mitigating cardiovascular diseases risks, such as atherosclerosis and myocardial infarction, by improving dyslipidemia, inflammation, and vascular dysfunction (58–60). Therefore, Amlexanox could potentially reduce AF risk by dampening the inflammatory response. Balsalazide shows promise in inhibiting ulcerative colitis-related carcinogenesis through IL-6/STAT3 signaling pathway (61), though it has been linked to pericarditis in some studies (62, 63). Olopatadine, a hypertrophic cell stabilizer and histamine H1 receptor antagonist, clinically targets allergic reactions but lacks reports on cardiovascular disease treatment (64–66). Methandriol and tibolone are both hormone drugs, which may mitigate inflammatory response and oxidative stress (9, 67), but carry hypertension risks (68). This study demonstrated that early amlexanox administration in acute sepsis mice could lessen inflammatory response and S100A12 expression, lowering AF risk and suggesting its efficacy in preventing and treating new-onset AF in sepsis. Consequently, amlexanox emerges as a promising candidate for treating new-onset AF in sepsis, potentially reducing sepsis progression and AF risk, thereby lowering mortality and enhancing the quality of life for patients with sepsis-induced AF.

This study, while contributing valuable insights, has its limitations. Firstly, although we observed a correlation between elevated S100A12 expression in serum and atrial tissue with increased susceptibility to new-onset AF in acute sepsis mice, we did not manipulate S100A12 gene to more definitively ascertain its relationship with new-onset AF in sepsis. Secondly, the exact mechanisms by which S100A12 influences the development of new-onset AF in sepsis warrant further exploration.

## 5 Conclusion

This study, through MR analysis, established sepsis as a potential independent risk factor for the onset and progression of AF, with no evidence of reverse causality. Furthermore, bioinformatics analysis and experimental evidence concerning sepsis and AF identified S100A12 as a potential pathological target for new-onset AF in sepsis, strongly linked to the inflammatory response. Amlexanox has shown effectiveness in preventing and treating new-onset AF in sepsis. These findings offer a fresh perspective on the pathogenesis of new-onset AF in sepsis and suggest potential targets and therapeutic options for its prevention and treatment.

## Data Availability

The datasets presented in this study can be found in online repositories. The names of the repository/repositories and accession number(s) can be found in the article/Supplementary Material.
